# Objective, Longitudinal Computed Tomographic Evaluation of the Metacarpal Condyles in Non-Lame Thoroughbred Racehorses †

**DOI:** 10.3390/ani16060973

**Published:** 2026-03-20

**Authors:** Vivien Putnoki, Danica Pollard, Sue Dyson, Koppány Boros, Annamaria Nagy

**Affiliations:** 1 Department and Clinic of Equine Medicine, University of Veterinary Medicine Budapest, Dóra Major, 2225 Üllő, Hungary; putnoki.vivien@univet.hu (V.P.); boros.koppany@univet.hu (K.B.); 2 Independent Researcher, The Rodhams, Rodham Road, Wisbech PE14 9NU, UK; drdee.pollard@gmail.com; 3 Independent Researcher, The Cottage, Church Road, Market Weston, Diss IP22 2NX, UK; sue.dyson@aol.com

**Keywords:** fetlock, metacarpophalangeal joint, third metacarpal bone, parasagittal groove, fan-beam CT, bone adaptation, Hounsfield Unit

## Abstract

There are limited data on sequential computed tomographic (CT) evaluation and objective CT assessment of the metacarpal condyles in Thoroughbred racehorses. This longitudinal study aimed to document changes in attenuation of the metacarpal condyles during the first two years of training and racing. Fan-beam CT examination of the metacarpophalangeal regions was performed on 40 non-lame Thoroughbred yearlings, and repeated four more times, approximately six months apart. Mean Hounsfield Unit (HU) measurements were obtained on sagittal reconstructions of the dorsal and palmar halves of the medial and lateral condyles and parasagittal grooves. One-way ANOVA with a post hoc Tukey’s Test was used to investigate differences between mean HU values over time at the different regions of interest. Multivariable mixed-effects linear regression models assessed the association between dorsal and palmar HU and potential explanatory variables. Mean HU increased significantly with training, especially during the first six months, with a maximal sequential mean increase found in the medial parasagittal groove (119.8 [95% confidence interval 85.3, 154.30], *p* < 0.001). Dorsal regions had higher HU than palmar regions, with the highest HU recorded in the dorsal aspect of the medial condyle at time 3 (mean HU 1120.1 ± 63.4). Condyles had higher HU than parasagittal grooves (*p* < 0.001), the palmar half of the right condyles had higher HU than the left (*p* = 0.045) and the dorsal aspect of the medial condyle had higher HU than the lateral (*p* < 0.001). An increasing number of race starts and higher body weight:height ratio were associated with higher HU (*p* < 0.001). The main limitation was the loss of horses to follow-up as the study progressed. In conclusion, density of most regions of the metacarpal condyles increased with time spent in training, reflecting adaption to racehorse training.

## Simple Summary

Lesions in the lower end of the cannon bone are a major cause of lameness and catastrophic injuries in Thoroughbred racehorses. Computed tomography (CT) is increasingly used in diagnosis, but longitudinal and objective data in non-lame horses are scarce. Knowledge of the findings in non-lame horses is essential for interpretation of images in lame horses. Forty Thoroughbred yearlings were examined using CT at five time points over two years, starting before race training. Attenuation, reflecting bone density, in specific regions of the front cannon bones was measured objectively. Attenuation increased with time spent in training, particularly in the first six months, reflecting adaptation to exercise. Differences were observed among regions: condyles had higher density than parasagittal grooves; the front half was denser than the back; and the front half of the inside condyle was denser than the outside. Increased body weight:height ratio and the number of race starts were associated with higher bone density. These changes in bone density reflect modelling of the bone associated with training exercise. This study demonstrated that CT can be used to monitor bone adaptation. Understanding normal adaptive changes is important for distinguishing them from pathological changes and it aids accurate interpretation of images of lame horses.

## 1. Introduction

Injuries of the metacarpophalangeal joint are common in Thoroughbred racehorses [1]. Lesions of the metacarpal condyles typically result from overloading and repetitive microtrauma often leading to reduced performance and lameness and may eventually result in fractures [1]. Catastrophic condylar fractures are common causes of racehorse fatalities worldwide [1,2]. Fractures are often associated with pre-existing pathological changes in the bone structure; recognising these changes at an early stage could potentially prevent more severe injuries [3].

Computed tomographic (CT) examination of the distal aspect of the limb can be performed with horses in a standing position and it facilitates early detection of structural osseous abnormalities [3,4,5]. Objective measurements of attenuation reflecting bone density allow the monitoring of adaptive changes that occur in response to training. The adaptation of the metacarpal condyles to exercise has been investigated using various imaging techniques in several studies [4,5,6,7,8,9]; most studies were performed on cadaver limbs [7,8,9]. A limited number of in vivo studies described changes in specific regions of the metacarpal condyles using CT examinations [4,5,6]. Alterations in the sagittal ridge of the third metacarpal bones (McIII) have been reported [10]; however, no longitudinal studies have investigated the adaptation of the metacarpal condyles using fan-beam CT. To the best of our knowledge, no previous research has documented CT changes in bone density of the distal aspect of the McIII with follow-up of longer than one year.

The aim of this longitudinal study was to describe changes in the metacarpal condyles in young non-lame Thoroughbred racehorses during their first two years of training and racing, using objective fan-beam CT measurements. In addition, the study aimed to investigate potential differences among different areas of the distal aspect of the McIII, and between the left and right forelimbs. It was hypothesised that density, as measured by Hounsfield Units (HU) in the metacarpal condyles, would increase with the progression of racehorse training and that there would be significant differences in density between regions of the metacarpal condyles, as well as between the right and left forelimbs due to the different loading.

## 2. Materials and Methods

Forty non-lame Thoroughbred yearlings with no history of metacarpophalangeal joint disease were included in the study. Racehorse owners and trainers were invited to participate; horses were selected in order of application. Signalment of horses (age, sex, body weight, height at withers) and training history were recorded [11]. Height at the withers was measured using a measuring stick and body weight was obtained using an electronic equine weighing scale. When enrolled, 27 horses had been engaged in some form of training (for 3–16 weeks, median 4, interquartile range [IQR] 4, 12); 26 were exercised in trot and one also in canter (no fast work) [11]. Computed tomographic examination of the metacarpophalangeal joint regions was performed prior to the start of racehorse training (time 0), and subsequently four times, approximately six months apart (times 1–4). Each time, diagnostic imaging was preceded by a clinical examination, as well as subjective (numerical grading system, ranging from 0 to 8) [12] and objective (Equinosis Q Lameness Locator, Columbia, SC, USA) lameness evaluations. At time 0, when many horses had been minimally handled, lameness examinations were performed at walk and trot, in a straight line on a hard surface. During subsequent examinations, a distal flexion test of each forelimb and lungeing in a circle, in trot on soft and hard surfaces, were also performed. If a horse showed forelimb lameness during these examinations, the pain was localised using diagnostic anaesthesia.

Computed tomographic examinations were performed with the horses in a standing position, under sedation using an intravenous combination of detomidine–hydrochloride (0.01 mg/kg IV, Domosedan, Orion Pharma, Budapest, Hungary) and butorphanol (0.02 mg/kg IV Nalgosed, Bioveta, Ivanovice na Hané, Czech Republic). The CT scanner used in the study, specifically designed for standing equine examinations (Qalibra CT System, Vet-DiCon, Zossen, Germany), is equipped with a 16-slice multidetector helical scanner with a 90 cm diameter (Canon Aquilion LB, Tokyo, Japan). The imaged forelimb was placed on the couch in a partially flexed, non- or semi-weightbearing position. Images were acquired with settings of 135 kV and 350 ms exposure time, a rotation time of 0.5 s, a slice thickness of 0.5 mm, and a 300 mm field of view. The CT scans were performed from the distal third of McIII to at least the junction of the proximal and distal halves of the proximal phalanx.

**Figure 1:**
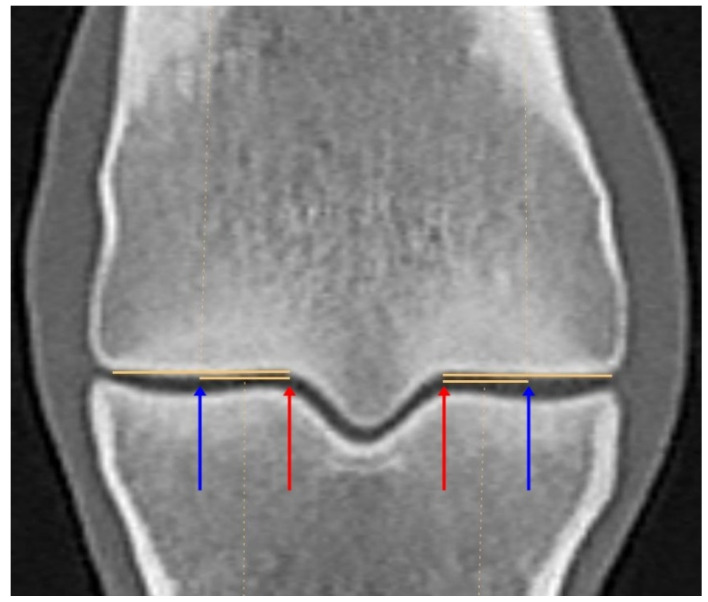
Frontal plane computed tomographic reconstruction of the distal epiphysis of the third metacarpal bone (medial is to the left), illustrating the locations of the sagittal reconstructions, used for Hounsfield Unit measurements planes. The upper orange horizontal lines indicate the mediolateral width of the medial and lateral condyles, while the lower orange lines represent one half of this width. The blue arrows indicate the mid-condylar measurement sites that were located halfway between the medial and lateral aspects of each condyle and the red arrows mark the parasagittal groove measurement sites.

**Figure 2:**
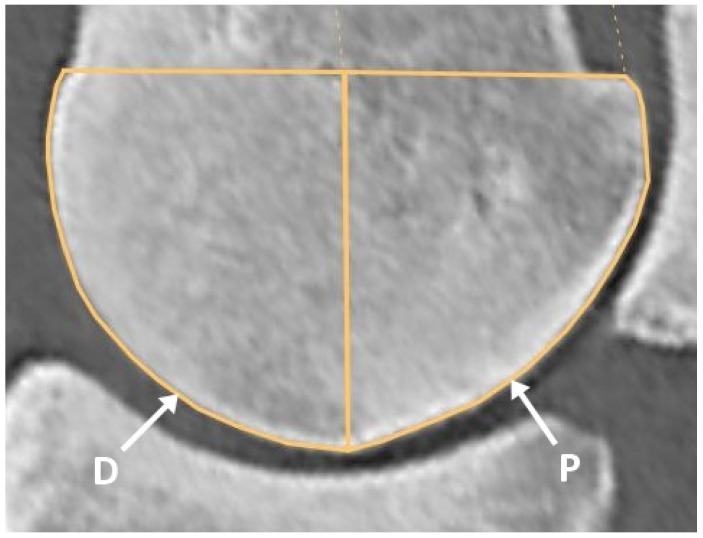
Sagittal plane computed tomographic reconstruction of the medial condyle of the third metacarpal bone, demonstrating the dorsal (D) and palmar (P) measurement regions, where mean Hounsfield Unit values were recorded. The midpoint of the line connecting the two most proximal points of the condyle was connected to its most distal point. The areas located dorsal and palmar to this line were then examined separately.

Computed tomographic images were analysed using Jivex DICOM Viewer Diagnostic Advanced 5.2 (Visus Health IT GmbH, Bochum, Germany) diagnostic software. The mean HU measurements were performed on images acquired in a bone algorithm, and reconstructed with a slab thickness of 0.3 mm. The frontal plane was aligned parallel to the vertically oriented axis of McIII. The sagittal plane was positioned to bisect the sagittal ridge of McIII along its midline, aligned parallel to the McIII axis, and the transverse plane was set perpendicular to it. On the sagittal reconstruction, the length of the line connecting the most proximal dorsal and palmar extents of the sagittal ridge was measured. The frontal plane was then positioned at two-thirds of this line. The location of the parasagittal grooves was identified on this frontal image. The medial and lateral condyle widths were measured starting from the parasagittal groove to the abaxial articular margin, and the midpoint of the measured section was determined. Hounsfield Unit measurements were performed by positioning the sagittal plane on four reference images: at the medial and lateral parasagittal grooves, as well as at the midpoints of the medial and lateral condyles (Figure 1). On the sagittal reconstructions, the midpoint of the line connecting the two most proximal points of the condyle was connected to its most distal point. The areas located dorsal and palmar to this line were then examined separately (Figure 2). The regions of interest were selected within the previously adjusted plane, and the mean HU values in each region were measured. Eight mean HU values were determined for each limb. Before starting the measurements, a repeatability test had been performed on five limbs, three times, at all measurement points. All measurements were obtained by a single assessor.

**Figure 3:**
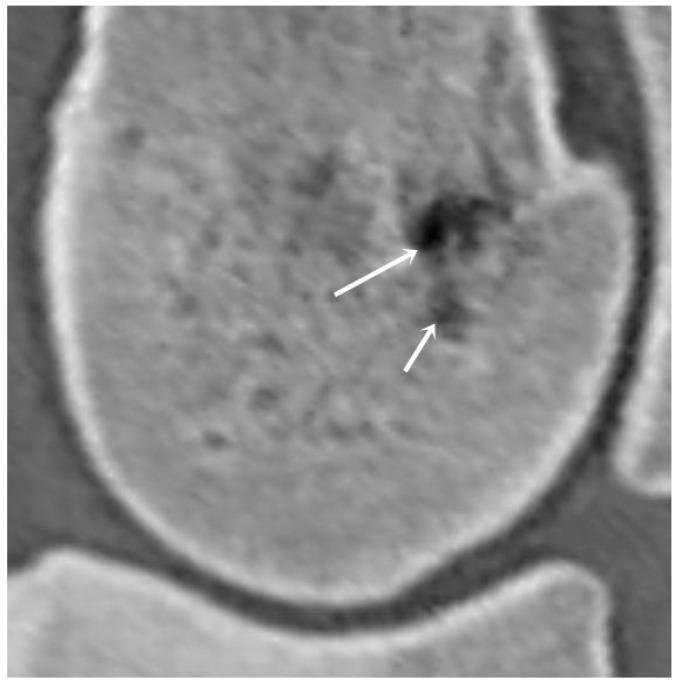
Sagittal plane computed tomographic reconstruction of the lateral parasagittal groove of a third metacarpal bone (dorsal is to the left). The arrows indicate vascular channels, which were documented and classified into three categories based on their size. The diameter of the proximal vascular channel is 3.1 mm, while that of the distal vascular channel is 1.9 mm.

The presence and number of vascular channels and hypoattenuating regions were also recorded in each examined region (Figure 3). The vascular channels were classified into three categories based on the size of their diameter (1.5–2 mm, 2.1–4 mm, >4 mm).

### Statistical Analysis

Data were collated in a Microsoft Excel spreadsheet (Office 365; Microsoft Corporation, Redmond, WA, USA). All statistical analyses were conducted using R Statistical Software (v4.3.1; R Foundation for Statistical Computing, Vienna, Austria. https://www.R-project.org, accessed on 20 August 2025).

Continuous variables (dorsal and palmar HU, age [months], body weight [kg] and height [cm]) were assessed for normality of distribution both visually via histograms and formally via the Shapiro–Wilk test. Additionally, a variable representing body weight:height ratio was calculated to consider the effect of both in a single variable. Normally distributed data (Shapiro–Wilk *p* > 0.05) were described using mean ± standard deviation while non-normally distributed and ordinal data (number of total starts, number of dorsal and palmar vascular channels and number of dorsal and palmar hypoattenuating areas) were described using medians with IQR. Categorical variables (sex [colt, filly, gelding], left or right forelimb and region [medial parasagittal groove, medial condyle, lateral parasagittal groove, lateral condyle]) were described using frequencies and proportions (%).

To initially examine whether mean palmar and dorsal HU measurements differed over examination time points, a one-way ANOVA with a post hoc Tukey’s Test for multiple comparisons was used to determine exactly at which time points mean palmar and dorsal HU measurements differed from each other within each region (medial condyle, medial parasagittal groove, lateral condyle, lateral parasagittal groove), with the assumption that measurements were not related. A one-way repeated measures ANOVA with post hoc pairwise paired *t*-tests could not be conducted because each horse had multiple measurements at each examination and loss to follow-up led to differing number of paired data across the time points.

Two individual multivariable mixed-effects linear regression models were used to examine the association between dorsal and palmar HU as the main outcomes and the potential explanatory variables already described. These explanatory variables were included as fixed effects in the model while horse (a unique identifier ID for each horse) was included as a random effect. This was because the data contained repeated observations, leading to a lack of independence between observations belonging to the same horse. In this way the model was adjusted to deal with this lack of independence by accounting for any similarities between observations belonging to the same horse. The ‘lme4’ package [13] was used to fit the models using restricted maximum likelihood (REML) and *t*-tests based on Satterthwaite’s method. Initially univariable mixed-effects linear regression models were used to test the association between dorsal and palmar HU and each explanatory variable individually. The multivariable models were built using backward elimination by initially including all preselected variables from the univariable models (where *p* < 0.20), with exclusion of non-significant variables, using the automated step function of the ‘lmerTest’ package based on least squares means and their differences for the fixed components of the model to select the final best fitting models for the data [14]. Results of the final models based on the automated selection were also manually checked to ensure the most biologically plausible variables were included in the final models. The relationship between variables likely to be closely correlated (e.g., age and examination number, and age and number of total starts) was assessed using Spearman’s rank correlation coefficient. To avoid multicollinearity, where two variables were highly correlated (≥0.8), the more biologically plausible variable was selected for inclusion in the model. The statistical significance for the final multivariable models was set at *p* ≤ 0.05.

## 3. Results

A total of 40 Thoroughbred yearlings (26 colts and 14 fillies) from ten racehorse trainers were included at time 0. Signalment and examination timings for horses at each examination timepoint are presented in Table 1. Some horses were lost to follow-up during the study period, due to withdrawal from training and/or discontinuation of participation at the request of the owners.

### 3.1. Dorsal and Palmar Hounsfield Unit Measurements

In the preliminary repeatability study of HU measurements, the coefficient of variance was <2%, which was considered acceptable.

**Figure 4:**
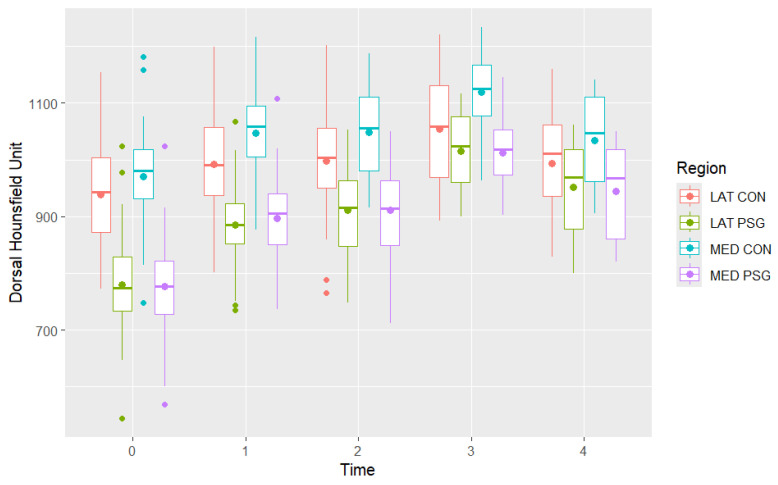
Distribution of total mean dorsal Hounsfield Unit measurements in the medial condyle (MED CON), medial parasagittal groove (MED PSG), lateral condyle (LAT CON) and lateral parasagittal groove (LAT PSG) in the metacarpophalangeal joints of Thoroughbred racehorses that were first examined as yearlings (time 0 n = 40) and then four more times at approximately six-month intervals (time 1 n = 31, time 2 n = 23, time 3 n = 13, time 4 n = 8). Boxes represent the median and interquartile range, whiskers the range and coloured circles the outliers.

**Figure 5:**
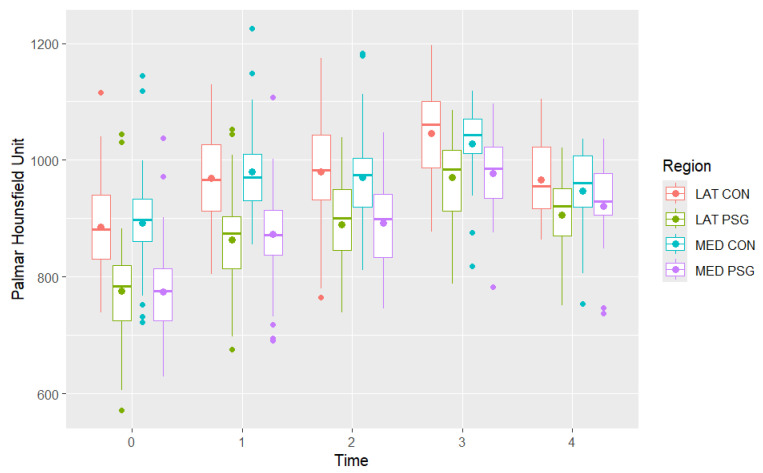
Distribution of total mean palmar Hounsfield Unit measurements in the medial condyle (MED CON), medial parasagittal groove (MED PSG), lateral condyle (LAT CON) and lateral parasagittal groove (LAT PSG) in the metacarpophalangeal joints of Thoroughbred racehorses that were first examined as yearlings (time 0 n = 40) and then four more times at approximately six-month intervals (time 1 n = 31, time 2 n = 23, time 3 n = 13, time 4 n = 8). Boxes represent the median and interquartile range, whiskers the range and coloured circles the outliers.

Descriptions of the mean dorsal and palmar HU measurement at each timepoint are presented in Table 2 and Figure 4 and Figure 5. The highest HU value was recorded in the dorsal aspect of the medial condyle at time 3, while the lowest HU value was recorded in the palmar aspect of the medial parasagittal groove at time 0. The highest dorsal HU values were recorded in the medial condyle and the lowest in the medial and lateral parasagittal grooves at all time points. The highest palmar HU values were recorded in the medial and lateral condyles and the lowest in the medial and lateral parasagittal grooves at all time points.

**Figure 6:**
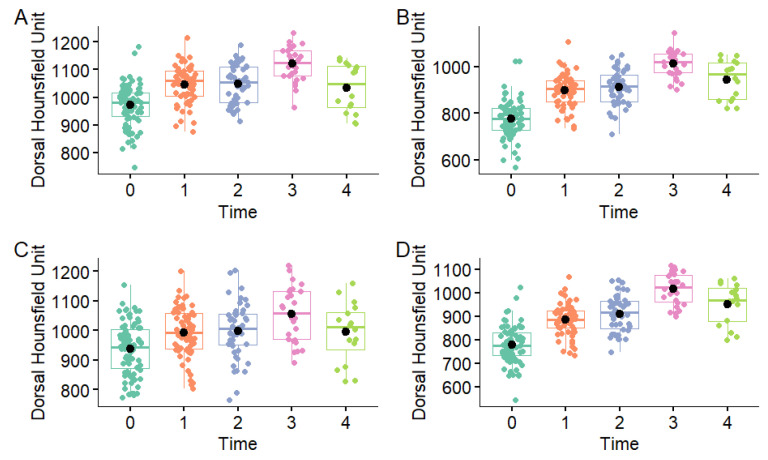
Distribution of total mean dorsal Hounsfield Unit measurements in the medial condyle (**A**), medial parasagittal groove (**B**), lateral condyle (**C**) and lateral parasagittal groove (**D**) in the metacarpophalangeal joints of Thoroughbred racehorses that were first examined as yearlings (time 0 n = 40) and then four more times at approximately six-month intervals (time 1 n = 31, time 2 n = 23, time 3 n = 13, time 4 n = 8). Boxes represent the median and interquartile range, whiskers the range and coloured circles the individual data points including outliers.

**Figure 7:**
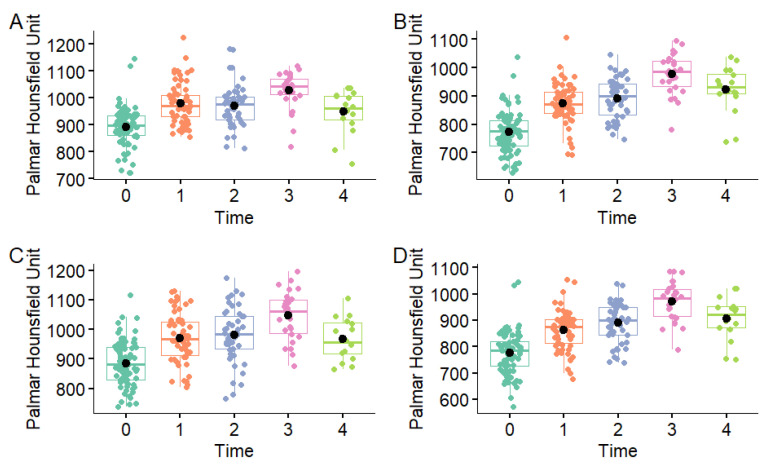
Distribution of total mean palmar Hounsfield Unit measurements in the medial condyle (**A**), medial parasagittal groove (**B**), lateral condyle (**C**) and lateral parasagittal groove (**D**) in the metacarpophalangeal joints of Thoroughbred racehorses that were first examined as yearlings (time 0 n = 40) and then four more times at approximately six-month intervals (time 1 n = 31, time 2 n = 23, time 3 n = 13, time 4 n = 8). Boxes represent the median and interquartile range, whiskers the range and coloured circles the individual data points including outliers.

**Figure 8:**
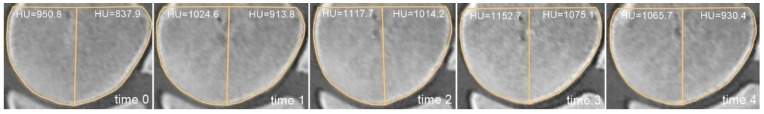
Sagittal computed tomographic reconstruction of the same lateral condyle of the third metacarpal bone, demonstrating changes in Hounsfield Unit (HU) values measured in the dorsal and palmar regions at five examinations approximately six months apart. The outlined regions of interest represent the dorsal (left) and palmar (right) halves of the lateral condyle. The measured HU values increased at times 1–3 compared with the previous examinations and decreased at time 4.

Initially, assuming independence between dorsal and palmar HU values at different time points, a one-way ANOVA showed a significant effect of examination time on mean dorsal and palmar HU values in all regions (*p* < 0.001) (Figure 6, Figure 7 and Figure 8). The dorsal HU at the first examination (time 0) was significantly lower than at all subsequent examinations in all regions, except for in the lateral condyle where the difference in mean dorsal HU measurements between the first (time 0) and fifth examination (time 4) was not significant (Table S1). The palmar HU at the first examination (time 0) was significantly lower than at all subsequent examinations in all regions, except for in the medial condyle where the difference in mean palmar HU measurements between the first (time 0) and fifth examination (time 4) was not significant (Table S2).

### 3.2. Multivariable Models

Table S3 contains the univariable models of the association between dorsal and palmar HU and explanatory variables. Age and examination number were strongly correlated (Spearman’s rho = 0.96, *p* < 0.001) as well as age and the number of total starts (Spearman’s rho = 0.83, *p* < 0.001). Examination time was also strongly correlated with number of total starts (Spearman’s rho = 0.85, *p* < 0.001). The number of total starts was selected for inclusion in the multivariable models as it was the most biologically plausible variable, showing the effects of exercise on dorsal HU. Body weight:height ratio replaced individual body weight and height variables where applicable as both body weight and height were strongly associated with dorsal HU (*p* < 0.001) at the univariable stage as well as being moderately correlated (Spearman’s rho = 0.50, *p* < 0.001).

The final multivariable models of variables associated with mean dorsal and palmar HU as the main outcome are presented in Table 3.

Dorsal HU measurements were higher on the medial side compared with the lateral side (*p* < 0.001), but lower in the parasagittal grooves compared to the condyles (*p* < 0.001). For each additional start, the total mean dorsal HU increased by 10.3 (*p* < 0.001); for each unit increase in body weight:height ratio, the total mean dorsal HU increased by 160.5 (*p* < 0.001).

Palmar HU measurements were lower in the parasagittal grooves compared with the condyles (*p* < 0.001) but there was no difference between medial and lateral sides. However, mean palmar HU measurements were higher in the right forelimb vs. the left forelimb (*p* = 0.04). For each additional start, the total mean palmar HU increased by 9.7 (*p* < 0.001); for each unit increase in body weight:height ratio, the total mean palmar HU increased by 154.9 (*p* < 0.001).

There were no dorsal hypoattenuating areas and only four palmar regions (1.7%) were identified in a total of 230 limbs analysed. The number of vascular channels was low in all size categories. Dorsal channels were limited to six observations in the 1.5–2 mm group (2.6%), three in the 2.1–4 mm group (1.3%), and only one exceeding 4 mm (0.4%). In the 1.5–2 mm category, one palmar channel was detected in 15 limbs (6.5%), two in two limbs (0.9%), and three in one limb (0.4%). In the 2.1–4 mm category, one channel was recorded in 25 limbs (10.9%) and two in one limb (0.4%). Channels greater than 4 mm were seen in only one limb (0.4%). Although some of these variables appeared to show associations in the univariable analysis, none remained meaningful in multivariable modelling. Consequently, because of their low frequency and limited analytical value, vascular channel counts and hypoattenuating areas were excluded from the final statistical analysis.

## 4. Discussion

In agreement with our hypothesis, a significant increase in HU was observed at times 1 and 3, compared with previous examinations. The increase was most pronounced between times 0 and 1, during the first six months of training. This agrees with the previous literature [4,10]. However, significant changes were not detected between every time point and in every region. Between times 1 and 2, no significant change was observed at any location. Between times 2 and 3, a significant increase was seen in all regions, except the dorsal part of the lateral condyle. Between times 3 and 4, no significant change or a significant decrease was documented. These patterns suggest that the greatest adaptive response occurs during the early phases of training. Times 2 and 4 occurred in late autumn or winter, when most horses were undergoing light training due to their winter rest period. Similar results were observed in the sagittal ridge of the McIII and in the proximal sesamoid bones in the same racehorse population [10,15]. For dorsal measurements, variability among horses was greater at time 0 compared with subsequent examinations; however, interpretation of this finding is limited by the small sample size.

Significant differences in HU were observed between anatomical regions. Comparing the condyles and the parasagittal grooves, radiodensity was significantly higher in the condyles than in the parasagittal grooves in both the palmar and the dorsal halves. This suggests that the condyles are exposed to higher loads, which likely stimulates increasing subchondral bone thickness and trabecular bone mineralization as part of the adaptive modelling process [6,16,17]. It is in concordance with the findings of a previous similar study based on cone-beam CT measurements in young Thoroughbreds, which documented that subchondral bone densification was more extensive in the condyles compared with the parasagittal grooves [4]. Additionally, dorsal HU values were consistently higher than palmar values, particularly in the condyles, indicating regional differences in load distribution and microarchitectural response during training. A previous study investigated bone stress distribution during impact on the distal end of the McIII, using finite-element analysis and documented the highest impact stresses in the dorsal region of the medial condyle [18]. The results of the current study are consistent with observations of the sagittal ridge of McIII in the same horse population [10], but contrast with some earlier studies that documented higher density in the palmar regions of the distal aspect of McIII [8,19]. The differing results might be explained by variations in training regimens and the type of surface on which the horses worked; differences in the risk of certain types of injuries on various track surfaces have been documented [20]. In Firth et al.’s study, horses worked on turf and sand racetracks [19], whereas the horses examined in the current study were trained on dirt and raced on dirt and turf. Differences in the type of surface on which the horses worked may have contributed to the discrepancies among studies; however, given the partial overlap in training and racing surfaces, this factor alone is unlikely to fully explain the observed differences. In addition, the studies examined different racehorse populations and were based on relatively small sample sizes (n = 14 [19]), which should be considered when interpreting and comparing the results.

Lateromedial asymmetry was detected in the dorsal regions of interest; the medial side had significantly higher HU compared with the lateral side. According to biomechanical analyses, the medial condyle has a greater contact area compared with the lateral condyle under impact loading [21]. Moreover, larger mean pressures and peak pressures were detected on the medial contact areas of the metacarpophalangeal joint compared with laterally [22]. These factors likely explain the greater density in the medial side.

A significant difference was observed between the right and left forelimbs. The mean HU values in the palmar regions of the right forelimb were higher than those of the left. This can be attributed to the racing and training direction. In Hungary, horses typically run clockwise in gallop races and they perform fast work predominantly on the right rein with the right forelimb leading. During gallop, the leading forelimb bears the highest loading [23,24]. Although the difference was statistically significant, it was minimal (1% difference between left and right forelimbs) and should not be overinterpreted.

The results of the current longitudinal, in vivo study align with previous cadaveric and in vivo studies on exercise-induced changes in the subchondral bone. Cadaver studies have demonstrated increased subchondral bone density in specific areas of the metacarpal condyles [7,8,9,25]. Similarly, Riggs and Boyde reported higher bone density in the distal aspect of McIII of exercised Thoroughbreds compared with the non-exercised horses, although these assessments lacked longitudinal follow-up [6]. In vivo studies using standing cone-beam CT confirmed exercise-induced densification in specific regions of the fetlock (medial and lateral mid-condyles and parasagittal grooves in the McIII; medial and lateral mid-fovea, medial and lateral ridge and sagittal groove in the proximal phalanx), but these studies were limited to a 12-month follow-up period [4,5].

A positive association was found between the number of race starts and HU values, suggesting that cumulative high-speed loading also contributes to bone adaptation. This aligns with previous studies in which increased exercise was linked to increased densification of the subchondral bone [5,6]. The body weight:height ratio was also positively correlated with HU values. Horses with higher ratios may exert relatively greater forces on joint surfaces, thus stimulating more pronounced subchondral modelling [26].

Being aware of adaptive changes present in non-lame horses is critically important for accurate interpretation of images of lame horses. To recognise pathological alterations, it is essential to understand the extent of physiological adaptation in horses at different stages of training. Numerous studies have focused on the distal epiphysis of the McIII [4,5,6,7,8,9,10,11,25,26,27,28,29,30]. Evaluation of the condyles and parasagittal grooves is of particular importance for risk assessment in condylar pathologies, because these regions are subjected to repeated high-magnitude mechanical loading during race training, which can result in the accumulation of microdamage within subchondral bone when the damage exceeds the capacity for repair [18]. In response, increased bone modelling and local densification may occur; however, this adaptive change may increase bone stiffness, predisposing the bone to fatigue-related injuries [18]. This longitudinal study demonstrates the ability of fan-beam CT to objectively monitor subchondral bone adaptation during race training. Recognising changes in bone density may aid in early identification of maladaptive modelling or overuse, potentially enabling intervention before clinical injury or fracture occurs. The marked differences between condylar and parasagittal groove regions, as well as between limbs and sides, emphasise the need for region-specific interpretation of imaging findings in clinical practice.

Although calibration phantoms were not used before each CT scan, several studies have demonstrated that HU values remain stable over extended periods without the need for repeated calibration [31,32,33,34]. Therefore, the absence of a calibration phantom is unlikely to have introduced significant bias or affected the overall validity and reproducibility of the HU measurements in this study.

In the current study, mean HU values were measured within predefined regions of interest rather than quantifying the linear extent of areas with greatest attenuation, as described by Ciamillo et al. [4]. This choice of methodology was based on several considerations. Areas of greatest attenuation do not have a sharp demarcation line, making it difficult to define the limits objectively. Using the current methodology, the HU measurements incorporated both the subchondral bone plate and the subchondral trabecular bone, thereby providing a more comprehensive assessment of adaptive changes. A similar method had been applied successfully in the same racehorse population for evaluating densification of the sagittal ridge [10]. Comparison of linear measurements and region of interest analysis merits further investigation.

### Limitations

Some horses were lost to follow-up, which may have introduced selection bias; however, to our knowledge no horses left the study due to condylar pathology. Furthermore, the loss of horses reduced statistical power at later time points. Training and racing regimens were not standardised, which may have influenced the results. The findings observed in this population may not be representative of racehorses trained under different conditions or on different surfaces.

## 5. Conclusions

The mean HU values of most regions of the metacarpal condyles increased with time spent in training, reflecting adaptation to racehorse training. The greatest increase occurred during the first six months of training, likely because the difference in loading intensity was the most pronounced between time 0 and time 1. Regional variations in condylar response suggest that the dorsal aspect of the medial condyle is subjected to the highest loads in the studied population.

## Figures and Tables

**Table 1:** Signalment and examination timings for 40 Thoroughbred racehorses that were first examined as yearlings (time 0, n = 40) and then four more times at approximately six-month intervals (time 1 n = 31, time 2 n = 23, time 3 n = 13, time 4 n = 8).

		Time 0 (n = 40)	Time 1 (n = 31)	Time 2 (n = 23)	Time 3 (n = 13)	Time 4 (n = 8)
Sex	Colt	26 (65.0%)	19 (61.3%)	12 (52.2%)	7 (53.8%)	3 (37.5%)
	Filly	14 (35.0%)	12 (38.7%)	8 (34.8%)	5 (38.5%)	2 (25.0%)
	Gelding	0 (0.0%)	0 (0.0%)	3 (13.0%)	1 (7.7%)	3 (37.5%)
Time since previous examination (days)	Median	-	194	198	213	175
	Interquartile range	-	182.0, 202.5 168.0, 237.0	194.5, 203.5 171.0, 253.0	183.0, 217.0 154.0, 238.0	167.0, 203.5 160.0, 226.0
Age (months)	Median	21.0	27.0	34.0	39.0	46.0
	Interquartile range	20.0, 22.0	26.0, 28.0	32.0, 35.0	38.0, 41.0	44.5, 47.5
Body weight (kg)	Median	440.0	458.0	460.0	440.0	450.0
	Interquartile range	402.5, 462.5	420.0, 476.0	420.0, 485.0	430.0, 453.0	445.0, 488.0
Height (cm)	Median	155.0	156.0	158.0	158.0	159.5
	Interquartile range	151.5, 157.0	154.0, 160.0	155.0, 161.0	156.0, 160.0	158.0, 161.0
Body weight:height ratio	Median	2.8	2.9	2.9	2.8	2.9
	Interquartile range	2.6, 3.0	2.7, 3.0	2.7, 3.1	2.7, 2.9	2.7, 3.0

**Table 2:** Total mean (±standard deviation) dorsal and palmar Hounsfield Unit (HU) measurements in four regions of the metacarpophalangeal joints of Thoroughbred racehorses that were first examined as yearlings (time 0 n = 40) and then four more times at approximately six-month intervals (time 1 n = 31, time 2 n = 23, time 3 n = 13, time 4 n = 8).

	**Medial Condyle**		**Medial Parasagittal Groove**	
**Examination Time**	**Dorsal HU**	**Palmar HU**	**Dorsal HU**	**Palmar HU**
0	971.0 ± 73.9	892.8 ± 72.1	777.3 ± 79.4	773.9 ± 74.4
1	1046.8 ± 67.2	980.4 ± 75.1	897.1 ± 69.7	872.8 ± 71.3
2	1048.0 ± 69.4	971.0 ± 79.7	911.0 ± 75.5	891.9 ± 71.9
3	1120.1 ± 63.4	1028.2 ± 69.2	1012.2 ± 57.6	977.4 ± 71.8
4	1033.6 ± 87.3	947.7 ± 80.2	944.6 ± 82.5	921.8 ± 85.4
	**Lateral condyle**		**Lateral parasagittal groove**	
	**Dorsal HU**	**Palmar HU**	**Dorsal HU**	**Palmar HU**
0	938.3 ± 87.6	885.2 ± 75.4	779.0 ± 77.7	775.2 ± 79.8
1	992.8 ± 83.6	969.1 ± 82.8	885.3 ± 67.7	863.4 ± 76.1
2	998.1 ± 95.1	979.9 ± 93.0	910.8 ± 76.6	889.3 ± 75.2
3	1054.6 ± 94.8	1046.0 ± 83.3	1015.9 ± 67.2	970.5 ± 75.2
4	993.4 ± 101.9	967.0 ± 70.7	951.8 ± 87.7	906.7 ± 81.5

**Table 3:** Final multivariable mixed-effects linear regression models of variables associated with dorsal and palmar Hounsfield Unit measurements as the main individual outcomes, with horse as a random effect, using data from Thoroughbred racehorses that were first examined as yearlings (time 0 n = 40) and then four more times at approximately six-month intervals (time 1 n = 31, time 2 n = 23, time 3 n = 13, time 4 n = 8).

**Outcome—Dorsal Hounsfield Unit**				
**Variable**	**Estimate**	**Standard Error**	***t*** **-Value**	***p*** **-Value**
*Side*				
Lateral	Reference			
Medial	23.93	4.63	5.17	<0.001
*Region*				
Condyle	Reference			
Parasagittal groove	−131.32	4.63	−28.36	<0.001
*Number of total starts*	10.25	0.81	12.62	<0.001
*Body weight:height ratio*	160.45	23.58	6.81	<0.001
**Outcome—Palmar Hounsfield Unit**				
**Variable**	**Estimate**	**Standard error**	***t*** **-value**	***p*** **-value**
*Region*				
Condyle	Reference			
Parasagittal groove	−95.67	4.62	−20.70	<0.001
*Number of total starts*	9.74	0.81	12.02	<0.001
*Body weight:height ratio*	154.85	23.50	6.59	<0.001
*Limb*				
Left	Reference			
Right	9.29	4.62	2.01	0.045

## Data Availability

Raw data can be accessed using the following link: https://doi.org/10.5281/zenodo.19098444.

## Supplementary Materials

The following supporting information can be downloaded at: https://www.mdpi.com/article/10.3390/ani16060973/s1, Table S1: Pairwise comparisons between total mean dorsal Hounsfield Unit (HU) values measured in the four different regions in the metacarpophalangeal joints of Thoroughbred racehorses that were first examined as yearlings (time 0, n = 40) and then four more times, at approximately six months’ intervals (time 1 n = 31, time 2 n = 23, time 3 n = 13, time 4 n = 8). Table S2: Pairwise comparisons between total mean palmar Hounsfield Unit (HU) values measured in the four different regions in the metacarpophalangeal joints of Thoroughbred racehorses that were first examined as yearlings (time 0, n = 40) and then four more times, at approximately six months’ intervals (time 1 n = 31, time 2 n = 23, time 3 n = 13, time 4 n = 8). Table S3: Univariable mixed-effects linear regression model results with dorsal Hounsfield Unit (HU) as the outcome. LAT CON = lateral condyle, LAT PSG = lateral parasagittal groove, MED CON = medial condyle, MED PSG = medial parasagittal groove.

## Abbreviations

The following abbreviations are used in this manuscript:

HU	Hounsfield Unit
IQR	Interquartile range
