# Supplementary material for: Objective, Longitudinal Computed Tomographic Evaluation of the Metacarpal Condyles in Non-Lame Thoroughbred Racehorses
Source: Animals (Basel). 2026 Mar 20;16(6):973. doi: 10.3390/ani16060973 (PMC13023280; doi:10.3390/ani16060973)
Supplement: Supplementary file 1 [file animals-16-00973-s001.zip › supplementary table S3.pdf]

Table S3

Univariable mixed-effects linear regression model results with dorsal Hounsfield Unit (HU) as the outcome. LAT CON=lateral condyle, LAT PSG=lateral parasagittal groove, MED CON=medial condyle, MED PSG=medial parasagittal groove

**Variable**	**Estimate**	**Standard error**	**t value**	**p-value**
Limb				
Left	Reference			
Right	2.61	6.83	0.38	0.703
Region				
LAT CON	Reference			
LAT PSG	-109.10	7.22	-15.11	<0.001
MED CON	46.14	7.22	6.39	<0.001
MED PSG	-107.39	7.22	-14.88	<0.001
Side				
Lateral	Reference			
Medial	23.92	6.80	3.52	0.001
Region				
Condyle	Reference			
Parasagittal groove	-131.31	5.21	-25.17	<0.001
Examination number	40.65	2.87	14.18	<0.001
Sex				
F	Reference			
G	12.69	27.50	0.46	0.646
M	-47.58	22.14	-2.15	0.038
Number of total starts	10.87	1.13	9.59	<0.001

Age (days)	0.21	0.01	14.07	<0.001
Age (months)	6.19	0.44	14.06	<0.001
Weight (kg)	1.43	0.17	8.28	<0.001
Height (cm)	15.07	1.31	11.50	<0.001
Body weight:height ratio	162.08	32.27	5.02	<0.001
Dorsal vascular channels 1.5-2 mm	-75.23	43.28	-1.73	0.083
Dorsal vascular channels 2.1-4 mm	-16.49	60.65	-0.27	0.786
Dorsal vascular channels >4 mm	-199.95	108.95	-1.84	0.067
Palmar vascular channels 1.5-2 mm	-33.84	18.95	-1.79	0.075
Palmar vascular channels 2.1-4 mm	-42.68	20.00	-2.14	0.033
Palmar vascular channels >4 mm	170.65	104.90	1.63	0.104
Palmar hypoattenuating areas	-130.67	55.14	-2.37	0.018

Univariable mixed-effects linear regression model results with palmar HU as the outcome

**Variable**	**Estimate**	**Standard error**	**t value**	**p-value**

Limb				
Left	Reference			
Right	9.29	6.04	1.54	0.124
Region				
LAT CON	Reference			
LAT PSG	-97.61	7.23	-13.49	<0.001
MED CON	0.56	7.23	0.08	0.938
MED PSG	-93.17	7.23	-12.88	<0.001
Side				
Lateral	Reference			
Medial	2.50	6.05	0.41	0.679
Region				
Condyle	Reference			
Parasagittal groove	-95.67	5.11	-18.72	<0.001
Examination number	39.97	2.48	16.14	<0.001
Sex				
F	Reference			
G	22.61	25.49	0.89	0.378
M	-48.81	21.03	-2.32	0.026
Number of total starts	10.17	1.00	10.19	<0.001
Age (days)	0.20	0.01	16.07	<0.001
Age (months)	6.09	0.38	16.05	<0.001

Weight (kg)	1.48	0.15	9.64	<0.001
Height (cm)	15.85	1.14	13.94	<0.001
Body weight:height ratio	163.88	29.14	5.62	<0.001
Dorsal vascular channels 1.5-2 mm	-39.29	38.29	-1.03	0.305
Dorsal vascular channels 2.1-4 mm	-44.16	53.56	-0.82	0.410
Dorsal vascular channels >4 mm	-112.17	96.60	-1.16	0.246
Palmar vascular channels 1.5-2 mm	-46.81	16.70	-2.80	0.005
Palmar vascular channels 2.1-4 mm	-14.46	17.72	-0.82	0.415
Palmar vascular channels >4 mm	98.08	92.75	1.06	0.291
Palmar hypoattenuating areas	-84.20	48.92	-1.72	0.086
