# Supplementary material for: Objective, Longitudinal Computed Tomographic Evaluation of the Metacarpal Condyles in Non-Lame Thoroughbred Racehorses
Source: Animals (Basel). 2026 Mar 20;16(6):973. doi: 10.3390/ani16060973 (PMC13023280; doi:10.3390/ani16060973)
Supplement: Supplementary file 1 [file animals-16-00973-s001.zip › supplementary table S2.pdf]

Table S2

Pairwise comparisons between total mean palmar Hounsfield Unit (HU) values measured in the four different regions in the metacarpophalangeal joints of Thoroughbred racehorses that were first examined as yearlings (time 0, n=40) and then four more times, at approximately six months' intervals (time 1 n=31, time 2 n=23, time 3 n=13, time 4 n=8).

Key: Diff: difference in means

lwr: lower 95% confidence interval of difference

upr: upper 95% confidence interval of difference

p adj: Tukey's adjusted p-value

Medial condyle

	Diff	lwr	upr	p adj
1-0	87.54	52.77	122.31	<0.001
2-0	78.19	40.16	116.22	<0.001
3-0	135.36	88.97	181.75	<0.001
4-0	54.85	-1.43	111.13	0.060
2-1	-9.35	-49.34	30.64	0.968
3-1	47.82	-0.20	95.83	0.052
4-1	-32.69	-90.31	24.94	0.525
3-2	57.17	6.75	107.59	0.017
4-2	-23.34	-82.98	36.31	0.819
4-3	-80.50	-145.80	-15.20	0.007

Medial parasagittal groove

	Diff	lwr	upr	p adj
1-0	98.88	64.65	133.12	<0.001
2-0	117.99	80.55	155.43	<0.001
3-0	203.48	157.80	249.15	<0.001
4-0	147.92	92.51	203.33	<0.001

	Diff	lwr	upr	p adj
2-1	19.11	-20.26	58.48	0.670
3-1	104.60	57.32	151.87	<0.001
4-1	49.03	-7.70	105.77	0.126
3-2	85.49	35.84	135.13	<0.001
4-2	29.92	-28.80	88.65	0.627
4-3	-55.56	-119.85	8.73	0.126

Lateral condyle

	Diff	lwr	upr	p adj
1-0	83.85	45.80	121.70	<0.001
2-0	94.62	53.01	136.23	<0.001
3-0	160.78	110.01	211.52	<0.001
4-0	81.81	20.23	143.39	0.003
2-1	10.77	-32.99	54.53	0.961
3-1	76.93	24.39	129.47	0.001
4-1	-2.04	-65.09	61.02	1.000
3-2	66.16	10.98	121.33	0.010
4-2	-12.81	-78.07	52.46	0.983
4-3	-78.96	-150.41	-7.51	0.022

Lateral parasagittal groove

	Diff	lwr	upr	p adj
1-0	88.16	52.09	124.23	<0.001
2-0	114.05	74.60	153.49	<0.001
3-0	195.31	147.19	243.43	<0.001
4-0	131.43	73.06	189.81	<0.001

	**Diff**	**lwr**	**upr**	**p adj**
2-1	25.89	-15.60	67.37	0.426
3-1	107.15	57.34	156.95	<0.001
4-1	43.27	-16.50	103.05	0.274
3-2	81.26	28.96	133.56	<0.001
4-2	17.39	-44.48	79.26	0.938
4-3	-63.87	-131.61	3.86	0.075
